# Comparison of Alginate Utilization Pathways in Culturable Bacteria Isolated From Arctic and Antarctic Marine Environments

**DOI:** 10.3389/fmicb.2021.609393

**Published:** 2021-01-27

**Authors:** Qian-Qian Cha, Xiu-Juan Wang, Xue-Bing Ren, Dong Li, Peng Wang, Ping-Yi Li, Hui-Hui Fu, Xi-Ying Zhang, Xiu-Lan Chen, Yu-Zhong Zhang, Fei Xu, Qi-Long Qin

**Affiliations:** ^1^State Key Laboratory of Microbial Technology, Marine Biotechnology Research Center, Shandong University, Qingdao, China; ^2^Department of Molecular Biology, Qingdao Vland Biotech Group Inc., Qingdao, China; ^3^College of Marine Life Sciences, Frontiers Science Center for Deep Ocean Multispheres and Earth System, Ocean University of China, Qingdao, China; ^4^Laboratory for Marine Biology and Biotechnology, Qingdao National Laboratory for Marine Science and Technology, Qingdao, China

**Keywords:** alginate, polar bacteria, enzymes, transporters, biogeographic distribution

## Abstract

Alginate, mainly derived from brown algae, is an important carbon source that can support the growth of marine microorganisms in the Arctic and Antarctic regions. However, there is a lack of systematic investigation and comparison of alginate utilization pathways in culturable bacteria from both polar regions. In this study, 88 strains were isolated from the Arctic and Antarctic regions, of which 60 strains could grow in the medium with alginate as the sole carbon source. These alginate-utilizing strains belong to 9 genera of the phyla *Proteobacteria* and *Bacteroidetes*. The genomes of 26 alginate-utilizing strains were sequenced and genomic analyses showed that they all contain the gene clusters related to alginate utilization. The alginate transport systems of *Proteobacteria* differ from those of *Bacteroidetes* and there may be unique transport systems among different genera of *Proteobacteria*. The biogeographic distribution pattern of alginate utilization genes was further investigated. The alginate utilization genes are found to cluster according to bacterial taxonomy rather than geographic location, indicating that the alginate utilization genes do not evolve independently in both polar regions. This study systematically illustrates the alginate utilization pathways in culturable bacteria from the Arctic and Antarctic regions, shedding light into the distribution and evolution of alginate utilization pathways in polar bacteria.

## Introduction

Brown algae, mainly distributed in cold coastal seawater, are multicellular organisms that constitute important biomass in coastal ecosystems. Alginate is a kind of polysaccharide that exists in the cell wall of brown algae, and it can make up 10 to 45% of the algal dry weight (Mabeau and Kloareg, 1987). Alginate is a linear polysaccharide composed of β-D-mannuronic acid (M) and α-L-guluronic acid (G) connected by 1,4-glucoside bond (Donati and Paoletti, 2009). It is an important carbon source to support the growth of marine microorganisms (Mabeau and Kloareg, 1987). Microorganisms are able to secrete alginate lyase (Aly) to degrade alginate into oligosaccharides and monosaccharides, which are further transported into the periplasm for further utilization (Wargacki et al., 2012). Sequences of alginate lyases are distributed in 12 polysaccharide lyase (PL) families in the Carbohydrate-Active enZYmes (CAZy) database, including the PL5, −6, −7, −14, −15, −17, −18, −31, −32, −34, −36, and −39 families^1^. In the 1960s, the alginate utilization pathway and related enzymes were firstly reported in *Pseudomonas* sp. (Preiss and Ashwell, 1962a,b). In the 1990s, the alginate utilization pathway in *Sphingomonas* sp. strain A1 was comprehensively studied. Four Aly genes and 1 reductase gene from strain A1 were heterogeneously expressed, and the crystal structures of these 5 enzymes were analyzed (Hashimoto et al., 2010; Takase et al., 2010; Yoon et al., 2000; Miyake et al., 2003; Hashimoto et al., 2000; Hashimoto et al., 2005). Until 2012, the enzymes and transporters involved in the alginate utilization pathway mainly from *Vibrio splendidus* 12B01 were cloned into *Escherichia coli*, which conferred *E. coli* the ability to degrade alginate to produce ethanol (Wargacki et al., 2012). In 2016, pectin degradation protein (KdgF) was confirmed to catalyze the conversion of unsaturated monouronates derived from pectin and alginate to linear ketonized form (Hobbs et al., 2016), which was previously thought to occur spontaneously.

Currently, more and more studies focused on the polysaccharide utilization loci (PULs) associated with alginate degradation using genomic sequencing (Sun et al., 2016; Kabisch et al., 2014; Mann et al., 2013; Xing et al., 2015). PULs are a cluster of adjacent genes that encode carbohydrate-active enzymes (CAZymes), carbohydrate transporters, and carbohydrate sensors/transcriptional regulators (Hemsworth et al., 2016; Foley et al., 2016; Martens et al., 2009). PULs were first defined in the *Bacteroidetes* bacteria (Bjursell et al., 2006),which are also widely distributed in the *Alpha-* and *Gamma-proteobacteria*. PULs associated with alginate degradation were first identified in marine *Bacteroidetes*, and the transcription of the genes located in PULs was strongly induced by alginate (Thomas et al., 2012). In this study, PULs associated with alginate degradation were referred to as alginate utilization gene clusters.

Two metabolic pathways of alginate utilization have been reported. In an alginate utilization pathway that is frequently found in marine bacteria, alginate is degraded to oligomers by Aly outside the cell, which are then transported to the periplasm through outer membrane proteins and are degraded into lower degree oligomers by Aly in the periplasm (Wargacki et al., 2012). The resultant oligomers are transported into the cytoplasm via oligoalginate transporters and are degraded to unsaturated monouronates by Aly in the cytoplasm (Wargacki et al., 2012). Unsaturated monouronate is converted to 4-deoxy-L-erythro-5-hexoseulose uronic acid (DEH) by pectin degradation protein (KdgF) (Hobbs et al., 2016). DEH reductase (DehR) reduces DEH into 2-keto-3-deoxy-D-gluconate (KDG) (Thomas et al., 2012), which is further phosphorylated by 2-dehydro-3-deoxygluconokinase (KdgK) to 2-keto-3-deoxy-6-phosphogluconate (KDPG). KDPG is converted to pyruvate and glyceraldehyde-3-phosphate via 2-dehydro-3-deoxyphosphogluconate aldolase (Eda) (Preiss and Ashwell, 1962b; Takase et al., 2010). Pyruvate and glyceraldehyde-3-phosphate are further assimilated through the glycolytic pathway (Supplementary Figure 1A). In addition, there are a few bacteria, such as *Sphingomonas* sp. strain A1, that use supersystem for alginate import/metabolism (Hashimoto et al., 2010). *Sphingomonas* sp. strain A1 can directly incorporate alginate polymer into the cytoplasm through a “superchannel” consisting of mouth-like pits on the cell surface, periplasmic binding proteins, and cytoplasmic membrane-bound ATP-binding cassette transporters. Subsequently, cytoplasmic Alys with different substrate specificities and action modes degrade the alginate to unsaturated monouronates, which are further converted into DEH. DEH is then assimilated through the ED pathway as described above.

Brown algae are cold-water algae and widely distributed in the Arctic and Antarctic regions. Metagenomic data analysis showed that bacteria with the ability to utilize alginates in the Arctic and Antarctic regions have high abundance and diversity (Matos et al., 2016). However, only a few culturable marine bacteria with alginate utilization ability have been isolated from the polar regions so far (Lavin et al., 2016; Xing et al., 2015). Systematic investigation of alginate metabolism pathways in bacteria from both polar regions is still lacking. Because the Arctic and Antarctic regions are long-distance separated, geographic isolation that impedes strain and gene transfer between both polar regions will be anticipated. How geographic isolation affects the evolution and transfer of alginate utilization genes in both polar regions is still elusive.

In this study, we isolated culturable bacteria that can utilize alginate from the Arctic and Antarctic marine environments, and sequenced the genomes of 26 alginate-utilizing strains. The alginate utilization pathways in these strains were analyzed and compared. Furthermore, the biogeographic distribution pattern of alginate metabolic genes in culturable bacteria from Arctic and Antarctic regions was investigated. The results shed light into the distribution and evolution of alginate utilization pathways in culturable bacteria from the polar regions.

## Materials and Methods

### Bacterial Isolation and Culturation

The total of 8 marine environmental samples, brown algae (BA1, BA2, BA3), penguin droppings (PD1, PD2), seawater (SWA1, SWA2), and marine sediment (SED), were collected from both polar regions (Supplementary Table 1). Before bacterial isolation, 8 samples were pre-treated according to the sample type. Penguin droppings samples (5 g) and sediment sample (5 g) were placed into Erlenmeyer flasks containing 100 ml sterile artificial seawater (ASW) prepared with Sigma sea salts (3%, w/v). Five liters of the seawater sample were filtered through a membrane filter with a pore size of 0.22 μm. After being cut into small pieces, the membrane samples were placed into Erlenmeyer flasks containing 100 ml sterile ASW. The pretreatment of brown algae sample was the same as that of membrane samples. The Erlenmeyer flasks were shaken for 3 h at 15°C with a speed of 180 rpm. The obtained suspensions from different pre-treatments were tenfold serially diluted to 10^–6^ dilution with sterile ASW. The diluted samples (200 μL) were spread on the minimal medium agar plates (per liter distilled water contained: 0.5 g NH_4_Cl, 30 g NaCl, 3 g MgCl_2_⋅6H_2_O, 2 g K_2_SO_4_, 0.2 g K_2_HPO_4_, 0.01 g CaCl_2_, 0.006 g FeCl_3_⋅6H_2_O, 0.005 g NaMoO_4_⋅7H_2_O, 0.004 g CuCl_2_⋅2H_2_O, 6 g Tris, and 15 g agar) supplemented with 0.5% (w/v) sodium alginate (Sigma-Aldrich). The agar plates were incubated at 15°C for 7–10 days. Colonies showing different morphologies on the plates were selected and purified by repeated streaking on TYS agar [per liter ASW contained: 5 g tryptone (Oxoid), 1 g yeast extract (Oxoid), and 15 g agar] at least 3 times. After purification, each strain was dotted on the minimal medium agar plate with sodium alginate and cultured at 15°C for 5 days. Then, the plates were stained with I_2_-KI to detect the appearance of a clear halo of depolymerization around a strain colony. Although this test might also reveal agar degradation, it was taken as a preliminary indicator of alginate degradation. To verify whether the strains with a clear halo on the plate could use alginate as the sole carbon source for growth, the strains were cultured in TYS broth [per liter ASW contained: 5 g tryptone (Oxoid), and 1 g yeast extract (Oxoid)] at 15°C to the logarithmic phase of growth, which were then centrifuged, washed, and resuspended in the minimal liquid medium. After the optical density at 600 nm (OD_600_) was adjusted to 1.0, 1% inoculum was inoculated into the minimal medium supplemented with 0.5% (w/v) sodium alginate (Sigma-Aldrich) as the sole carbon source and the minimal medium without sodium alginate (negative control). The inocula were cultured at 15°C with a shaking speed of 180 rpm and the OD_600_ of the culture was detected with Bioscreen C Microbiology reader (Oy Growth Curves Ab Ltd., Finland) to record the bacterial growth.

### Genome Sequencing and PCR

Genomic DNA of all strains was extracted using a DNA extraction kit (BioTeke, China). The libraries of 16 strains (No. 1 to No. 16) were qualified by the Agilent Technologies 2100 bioanalyzer and ABI StepOnePlus Real-Time PCR System (Supplementary Table 2). The qualified libraries were sequenced pair-end using HiSeq System (Illumina). The raw sequencing data was quality-filtered using the SOAPnuke (Chen et al., 2017). The clean reads were assembled using SOAPdenovo v2.04 (Li et al., 2008; Li et al., 2010) to get the draft genomes (Supplementary Table 2). The libraries of 10 strains (No. 17 to No. 26) were prepared according to the instructions from Pacific Biosciences, following the Preparing Multiplexed Microbial Libraries Using SMRTbell Express Template Prep Kit 2.0. Then, genomes of these 10 strains were sequenced on a PacBio Sequel platform and assembled using the HGAP 4 analysis process implemented in SMRT Link (V6.0.0.47841) (Chin et al., 2013) to get the complete genomes (Supplementary Table 2). The completeness and contamination of the obtained 26 genomes were assessed with CheckM (Parks et al., 2015). All the genomes were annotated using the RAST server^2^ (Aziz et al., 2008), and then manually verified and corrected. Polysaccharide lyase (PL) families of the alginate lyase genes were annotated by HMMER (v3.3.1) (Mistry et al., 2013). Taxonomy positions of strains were identified based on 16S rRNA gene sequence analyses. The 16S rRNA genes of the strains were PCR-amplified using primers 27F and 1492R (Weisburg et al., 1991). The general characteristics for draft or complete genome sequences of the 26 strains are shown in Supplementary Table 2. In addition, the genomes of 6 strains previously isolated from the polar region were downloaded for genomic analysis (Supplementary Table 3).

### Phylogenetic Analyses

Protein sequences of 5 functional genes (*aly*, *kdgF*, *dehR*, *kdgK*, and *eda*) and the 16S rRNA gene sequences were used to construct phylogenetic trees. Each strain contains 1–10 *aly* genes in its genome. There are totally 159 *aly* genes in the genomes of the 32 strains. Among them, most strains contain one or more *aly* genes belonging to the PL7 family. Then, 21 PL7 family *aly* gene sequences from 21 strains (only one *aly* gene from one strain) were selected to construct the phylogenetic tree. For other functional genes (*kdgF*, *dehR*, *kdgK*, and *eda*), the sequences in the alginate utilization gene clusters were selected to construct the phylogenetic trees. Sequences of the 16S rRNA genes and 5 functional genes were aligned using Muscle version 3.8.31 (Edgar, 2004). The phylogenetic trees based on the 16S rRNA gene sequences and protein sequences of the 5 functional genes were performed using MEGA 7.0 (Kumar et al., 2016) and reconstructed using the neighbor-joining and maximum-likelihood algorithms. Bootstrap values were calculated based on 1000 replicates.

## Results and Discussion

### Diversity Analyses of the Culturable Bacteria

**FIGURE 1:**
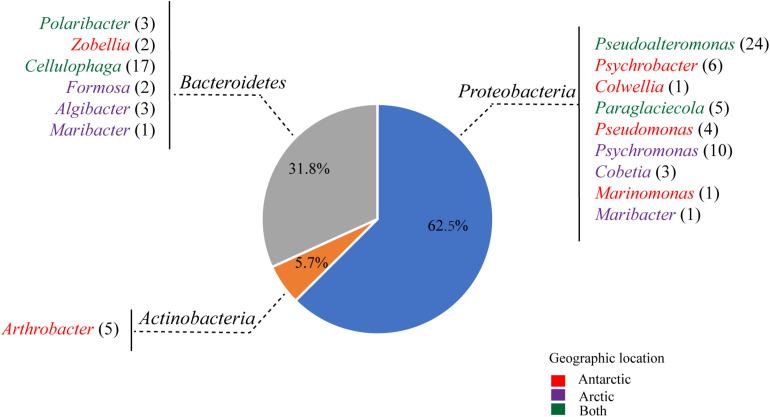
Abundances of the culturable bacteria isolated from 8 samples (PD1, PD2, SED, SWA1, SWA2, BA1, BA2, and BA3) from the Antarctic and Arctic marine environments. Colors indicate the original locations of the different bacterial genera: the Antarctic only (red); the Arctic only (purple), both of the Antarctic and Arctic (green).

Totally, 88 strains were selected from the agar plates containing alginate, of which 43 strains were from the Arctic and 45 strains from the Antarctic (Figure 1). These culturable strains belonged to 16 genera of the phyla *Proteobacteria* (62.5%), *Bacteroidetes* (31.8%), and *Actinobacteria* (5.7%) based on the phylogenetic analyses of their 16S rRNA genes. Among the 88 strains, 60 could grow in the minimal medium with alginate as the sole carbon source, which belonged to 9 genera of the phyla *Proteobacteria* (*Gammaproteobacteria*) and *Bacteroidetes* (*Flavobacteriia*): *Pseudoalteromonas* (22), *Cellulophaga* (16), *Psychromonas* (5), *Paraglaciecola* (5), *Cobetia* (3), *Polaribacter* (3), *Algibacter* (3), *Formosa* (2), and *Colwellia* (1). Among the four isolation sources, the abundance of alginate-degrading bacteria from brown algae is the highest (81.4%), followed by those from seawater (70%) and sediment (61.5%), and that from penguin droppings (25%) is the lowest (Supplementary Figure 2).

The metagenomics of sediments from four high-latitude coastal environments of both Hemispheres revealed that the sediments contain highly abundant and diverse bacterial assemblages with alginolytic potential, mainly belonging to the phyla *Proteobacteria* and *Bacteroidetes* (Matos et al., 2016). Martin et al. and Dong et al. screened alginate-degrading bacteria from French beach and Arctic brown algae, which belonged to the classes *Gammaproteobacteria* and *Flavobacteriia* of the phyla *Proteobacteria* and *Bacteroidetes* (Martin et al., 2015; Dong et al., 2012). Consistent with these reports, our results indicated that bacteria from the phyla *Proteobacteria* and *Bacteroidetes* play an important role in the alginate utilization in the both poles.

### Key Enzymes in the Alginate Utilization Pathway

According to the geographic location and bacterial taxonomy, 21 strains that could utilize alginate as the sole carbon source were used for genomic sequencing. In addition, the genomes of 5 alginate-utilizing strains (*Pseudoalteromonas* sp. C1, *Pseudoalteromonas* sp. C7, *Pseudoalteromonas* sp. C8, *Pseudoalteromonas* sp. SA25, and *Psychromonas* sp. SA13A) previously isolated from polar regions in our lab were also sequenced to investigate their alginate utilization pathway. The GC contents (%) of 18 *Proteobacteria* genomes are higher than 35%, and those of 8 *Bacteroidetes* genomes are lower than 35% (Supplementary Table 2). Validation of the genome assembly showed that the completeness of the 26 genomes are not less than 99% (Supplementary Table 2). The high completeness of genomes can ensure the correctness of genomic analyses.

We calculated the content of alginate utilization genes (all the alginate lyase genes in the genomes and related genes in the gene clusters) in the coding sequences (CDSs) of these bacteria. The average content of alginate utilization genes of the bacteria isolated from brown algae is 0.33%, 0.3% in those from sediment, 0.29% in those from penguin dropping, and 0.27% in those from seawater (Supplementary Table 2). However, *t*-test result indicates that there is no significant difference in the average content of alginate utilization genes between different isolated sources (*p* > 0.05).

The 26 strains could grow in the medium with alginate as the sole carbon source (Supplementary Figure 3), indicating that they all have a functional alginate utilization pathway. The assimilation from alginate polymers to glycolytic pathways requires the catalysis of 5 key enzymes: Aly, KdgF, DehR, KdgK, and Eda (Supplementary Figure 1A; Wargacki et al., 2012; Hobbs et al., 2016). We analyzed the distribution of the genes encoding these 5 key enzymes in the 26 alginate-utilizing strains. In addition, 6 polar-origin strains with published genomes and containing the *aly* genes are also included for comparison (Van Trappen et al., 2004; Kim et al., 2013; Kim et al., 2014; Li et al., 2017; Wang et al., 2017; Choi et al., 2018). Thus, there are 32 genomes used for analyses of alginate utilization pathways in bacterial strains isolated from the Arctic and Antarctic marine environments in this study.

**FIGURE 2:**
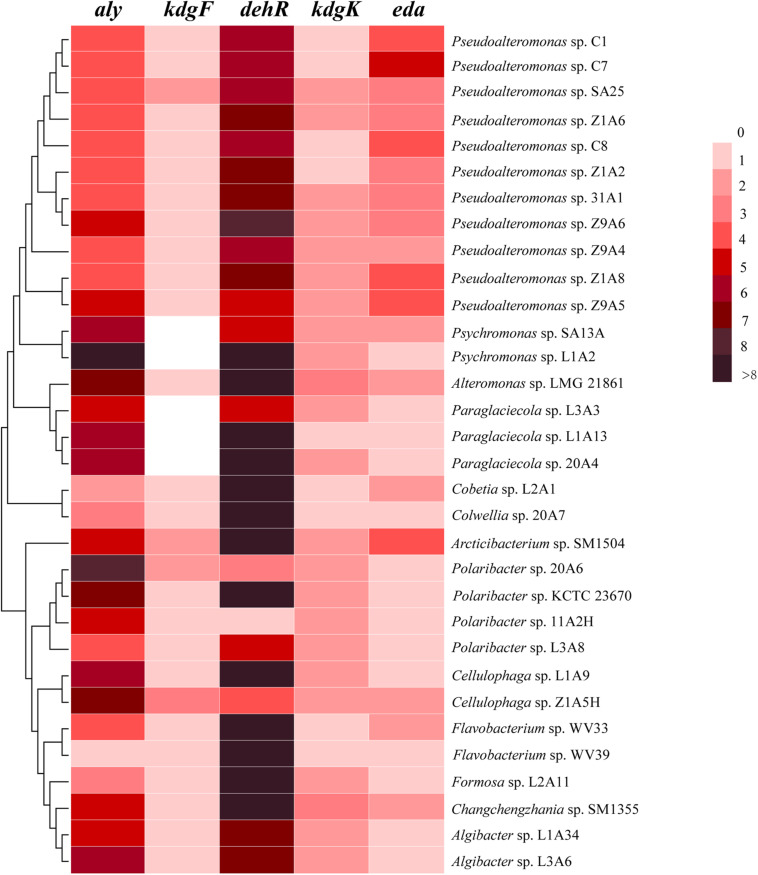
The number of homologs of the alginate utilization genes in the 32 genomes. *aly*, alginate lyases; *kdgF*, pectin degradation protein; *dehR*, DEH reductases; *kdgK*, 2-dehydro-3-deoxygluconate kinase; *eda*, 2-dehydro-3-deoxyphosphogluconate aldolase.

**FIGURE 3:**
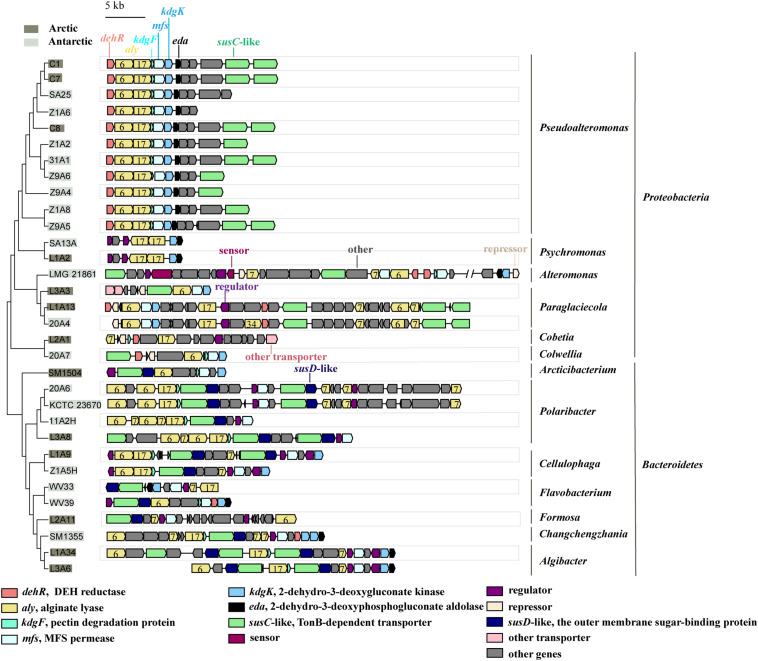
The alginate utilization gene clusters in the genomes of the polar bacteria. The gray-covered bacteria were isolated from the Arctic and the light-gray covered bacteria were isolated from the Antarctic. PL families are indicated by numbers.

All the genomes contain the genes encoding the 5 key enzymes involved in alginate utilization, except that the *kdgF* gene is absent from the genomes of 5 strains (Figure 2). These key genes tend to be present in the alginate utilization gene cluster (Figure 3 and Supplementary Figure 4). Aly is an important enzyme in the utilization of alginate. Each genome contains at least 1 *aly* gene, and 87.5% of the genomes contain 4 to 8 *aly* genes. There are 159 *aly* genes in the 32 genomes, which belong to PL6 (45/159), −7 (68/159), −12 (2/159), −17 (31/159), −18 (11/159), and −34 (2/159) families (Supplementary Table 4). So far, the reported PL12 family enzymes in CAZY database are only active to heparin and heparin-sulfate. Whether the 2 genes from the PL12 family annotated as Aly by RAST are active to alginate needs to be further verified. *Aly* genes from PL6, −7, and −17 are abundant in both *Proteobacteria* and *Bacteroidetes*, whereas *Aly* genes from PL18 and −34 are specific to *Proteobacteria* (Supplementary Table 4). Most of the *aly* genes (88/159) are clustered in the alginate utilization gene clusters, which belong to PL6, −7, and −17 (Figure 3). Except for the genomes of 2 *Psychromonas* strains and 3 *Paraglaciecola* strains, the other genomes all contain the *kdgF* gene, indicating that the *kdgF* gene is essential in the alginate utilization process of the alginate-utilizing strains. In the 5 *kdgF*-lacking strains, the conversion of unsaturated monouronate to DEH may be catalyzed by other enzymes or spontaneously occur (Takase et al., 2010). There are 2 types of genes encoding DehR family proteins, i.e., A-type *dehR* (encoding 3-hydroxyisobutyrate dehydrogenase) and B-type *dehR* (encoding 2-dehydro-3-deoxy-D-gluconate 5-dehydrogenase). All the 32 strains contain at least 1 type *dehR* gene in their genomes, but not all the *dehR* genes are present in the alginate utilization gene cluster. Among the 13 gene clusters of *Bacteroidetes*, 2 contain the *dehR* genes, both of which are B-type *dehR*. There are 16 gene clusters of *Proteobacteria* containing *dehR* genes, among which 11 gene clusters of *Pseudoalteromona*s spp. and *Colwellia* sp. 20A7 contain A-type *dehR* gene, 3 gene clusters of *Paraglaciecola* sp. L1A13, *Paraglaciecola* sp. 20A4, and *Cobetia* sp. L2A1 contain B-type *dehR* gene, and the gene cluster of *Alteromonas* sp. LMG 21861 contains 2 types of *dehR* genes. All the genomes have *kdgK* and *eda* genes. Altogether, except for 5 genomes that contain no *kdgF* gene, all the genomes contain the 5 key enzymes genes (*aly*, *kdgF*, *dehR*, *kdgK*, and *eda*) (Figure 2). This indicates that the alginate utilization pathway previously reported is widespread in the marine bacteria isolated from the polar regions.

### Transporters in the Alginate Utilization Gene Cluster

Transporters also play an important role in alginate utilization. Carbohydrate transport system includes the outer membrane transport system and the inner membrane transport system (McBride et al., 2009). The well-studied outer membrane transport system includes the outer-membrane sugar-binding protein (SusD-like protein) and TonB-dependent transporter (TBDT; SusC-like protein) that are located adjacently to each other, *susC-susD* pair. SusD-like protein specifically binds oligosaccharides (Tauzin et al., 2016; Koropatkin et al., 2009; Koropatkin et al., 2008; Bakolitsa et al., 2010; Phansopa et al., 2014), and TBDT provides a channel for oligosaccharides to enter the periplasm (Supplementary Figure 1B; Reeves et al., 1996; Della and Salyers, 1996a,b; Reeves et al., 1997). The inner membrane transport system mainly constitutes a group of permeases/transporters that transport oligosaccharides and monosaccharides from the periplasm into the cytoplasm (Martens et al., 2009). Major facility superfamily (MFS) permease is likely an inner membrane transporter that transports oligomers of alginate and unsaturated monouronates from the periplasm into cytoplasm (Supplementary Figure 1B; Grondin et al., 2017). Genes encoding transporters related to alginate utilization were reported to be mainly present in the alginate utilization gene clusters (Thomas et al., 2012; Kabisch et al., 2014; Grondin et al., 2017). Therefore, we mainly analyzed the kind of transporters present in the predicted alginate utilization gene clusters of the 32 strains.

Analyses of alginate utilization gene clusters show that all strains of *Bacteroidetes* have at least 1 *susC-susD* pair in their gene clusters (Figure 3). The *susC-susD* pair is the hallmark of PULs (Grondin et al., 2017). The outer membrane transport systems in *Proteobacteria* are different from those in *Bacteroidetes*. While the *susC*-like genes are found in the alginate utilization gene clusters of 74% strains of *Proteobacteria*, the *susD*-like gene is absent from all the gene clusters of *Proteobacteria* (Figure 3). This finding is consistent with previous report that *Proteobacteria* may obtain alginate utilization system from *Bacteroidetes* via horizontal gene transfer (HGT), but the SusD-like proteins seemed to be lost due to the incompatibility with the physiology of the *Proteobacteria* (Thomas et al., 2012). For the inner membrane transport system, all the alginate utilization gene clusters of *Bacteroidetes* and *Proteobacteria* contain *mfs* gene, except that the *mfs* gene is absent from the gene clusters of *Cobetia* sp. L2A1, *Psychromonas* sp. SA13A and *Psychromonas* sp. L1A2 (Figure 3).

These data show that the alginate utilization transport systems are relatively conserved in the *Bacteroidetes* strains isolated from polar regions. *Proteobacteria* strains may lose specific genes related to alginate transport system, such as *SusD*-like genes and *mfs* genes. This indicates that the *Proteobacteria* and *Bacteroidetes* have different alginate utilization transport systems form.

### Sensors, Regulators and Repressors in the Alginate Utilization Gene Clusters

The expression of all genes in a PUL is co-regulated and tightly controlled by the presence of the substrate, which is achieved by transcriptional regulatory system (Grondin et al., 2017; Dudek et al., 2020). In 2020, the first model of regulation for a PUL of alginate utilization in the marine bacterium *Zobellia galactanivorans* Dsij^*T*^ was proposed (Dudek et al., 2020). The AusR protein of GntR family binds to promoters via single, double or triple copies of the operator. After the addition of alginate, metabolic intermediates produced during the basic level degradation inhibit the formation of AusR/DNA complexes, thereby enhancing transcriptional inhibition. Although a regulation model of alginate utilization has been reported, our knowledge on the transcriptional regulation of alginate utilization is still limited, and more investigations are necessary.

To further explore the transcriptional regulatory systems of the alginate utilization gene clusters in marine bacteria, we analyzed the sensors, regulators and repressors in the 32 genomes (Figure 3). Sensors are absent from most gene clusters, except that 2 two-component systems (TCSs) are found in the gene cluster of strain LMG 21861. In addition to 2 TCSs, 29 regulators are identified in 32 gene clusters. These regulators belong to the GntR (16/29), LacI (8/29), TetR (3/29), and Crp (2/29) families. The regulators in the Crp family are specific to the genus *Cellulophaga*. There are 5 repressors in *Proteobacteria*. Three of them belong to the GntR family, and the others are not clearly classified. Interestingly, transcriptional regulators are not found in the gene clusters in *Pseudoalteromonas* by gene annotation. This suggests that *Pseudoalteromonas* may have a different transcriptional regulatory system for alginate utilization form other bacteria, which needs further investigation.

### The Biogeographic Distribution of Alginate Utilization Genes in Arctic and Antarctic Regions

The genomes of the 32 strains isolated from the polar regions all contain alginate utilization genes. Due to the long distance between the Arctic and Antarctic regions, geographic isolation might have influenced the transfer and evolution of these genes. If long-distance can limit the interchange of bacterial strains and gene transfers between the Arctic and Antarctic regions, HGT will happen only within local habitats, then each polar region will have its specific alginate utilization gene categories, and even develop different utilization pathways. Then the functional genes in the phylogenetic tree will cluster based on geographic locations.

The 16S rRNA gene is seldom subject to HGT and considered as a suitable material for reconstructing ancient phylogenetic relationships (Woese et al., 1990). The phylogenetic trees of the 16S rRNA gene sequences were used to evaluate the phylogenetic relationships of bacterial strains. Because the genes encoding the 5 key enzymes involved in the alginate utilization process are present in both polar regions, the phylogenetic trees based on the protein sequences of the 5 key enzymes were compared with the phylogenetic trees of the 16S rRNA gene sequences to investigate whether the alginate utilization genes are clustered based on geographic locations or based on the taxonomy of the strains where genes are from.

**FIGURE 4:**
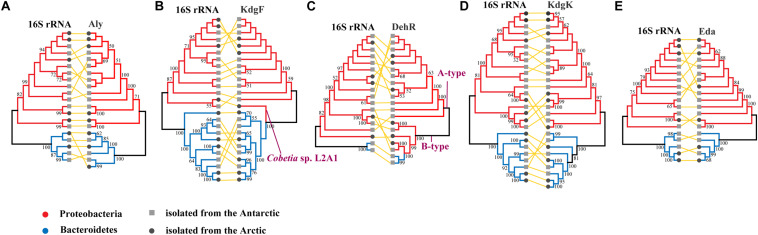
Comparison of the neighbor-joining phylogenetic trees constructed based on the protein sequences of 5 key enzymes of alginate utilization (the right) and the 16S rRNA gene sequences (the left). Bootstrap values (>50%) based on 1000 replicates are shown at nodes. The terminal in both trees represent the strain where the protein sequence and the 16S rRNA gene sequence are from. The same strain in both trees is linked with yellow line. The strains from *Proteobacteria* are marked in red, and the strains from *Bacteroidetes* are marked in blue. The strains isolated from Antarctic are marked in gray square, and the strains isolated from Arctic are marked in dark gray circle. The comparisons of Aly, KdgF, DehR, KdgK, and Eda are marked as **(A–E)**, respectively.

The comparison shows that there is no significant difference between the topographies of phylogenetic trees constructed using the alginate utilization genes and the 16S rRNA genes (Figure 4 and Supplementary Figure 5). The topography of the phylogenetic tree constructed using the Aly protein sequences is consistent with its phylogenetic tree of the 16S rRNA genes, which is divided into 2 branches according to bacterial taxonomy (*Proteobacteria* and *Bacteroidetes*) (Figure 4A and Supplementary Figure 5A). The phylogenetic tree based on protein sequences of KdgF is divided into 2 large branches, *Proteobacteria* and *Bacteroidetes*, with 1 exception that the KdgF from *Cobetia* sp. L2A1 of *Proteobacteria* clusters in the branch of *Bacteroidetes* in the neighbor-joining tree (Figure 4B and Supplementary Figure 5B). In addition to the clear separation between *Proteobacteria* and *Bacteroidetes*, *Pseudoalteromonas* are geographically clustered in the Aly and KdgF trees (Figures 4A,B and Supplementary Figures 5A,B). The phylogenetic tree of DehR is clustered by gene type, rather than by bacterial taxonomy. The A-type DehRs form 1 branch, and the B-type DehRs form another branch (Figure 4C and Supplementary Figure 5C). The topographies of the phylogenetic trees based on KdgK and Eda protein sequences are consistent with the phylogenetic trees of the 16S rRNA genes, and both phylogenetic trees are divided into 2 branches according to bacterial taxonomy (*Proteobacteria* and *Bacteroidetes*) (Figures 4D,E and Supplementary Figures 5D,E). Therefore, despite a few inconsistencies, phylogenetic analyses generally indicate that the alginate utilization protein sequences tend to cluster according to bacterial taxonomy rather than geographic location.

We further compared the protein sequence of the 5 key enzymes to investigate whether the isolation location affects the pair-wise sequence identities. When all the protein sequences of one specific functional gene derived from the Arctic region were compared to the protein sequences derived from the Antarctic region, the average sequence identity of all the pair-wise sequence identities was referred as between-pole protein sequence identity of one specific functional gene. When the protein sequences of one specific functional gene derived from the same polar region (Arctic or Antarctic) were compared to each other, the average sequence identity was referred as within-pole sequence identity of one specific functional gene.

The between-pole and within-pole sequence identities of the 5 key enzymes all showed no significant differences (*p* > 0.05, *t*-test) (Supplementary Figure 6), indicating that there is no significant difference in alginate utilization protein sequences between the Arctic and Antarctic. Thus, both the sequence identities and phylogenetic analyses showed that there is no significant geographic isolation that impede the transfer of the alginate utilization genes between both poles.

A possible explanation for this biogeographic distribution pattern of alginate metabolic genes was proposed. All the strains in this study were isolated from the marine environment, and the ocean is a contiguous aquatic environment. It is reported that different regions in the global surface ocean are connected through thermohaline circulation on the time scale of ∼1,000 to 2,700 years (DeVries and Primeau, 2011). However, it will take 10 s of millions of years for the HGT components to fully integrate into the metabolic network to function (Lercher and Pal, 2008). It appears that marine strains from different geographic locations can be well connected before the HGT components integrate into the metabolic network. In addition, plasmids, as the carrier of HGT, are widely found in marine bacteria. Plasmids with 100% sequence identity were detected from vast geographic distances (Petersen et al., 2019). Plasmids may integrate different gene sequences across vast phylogenetic and geographic distances, suggesting that marine bacterial communities are more connected than we thought. Therefore, although the Arctic and the Antarctic are geographically distant, there may be no geographic isolation for the transfer of the alginate utilization genes between both poles.

The driving forces for marine bacterial biogeography may be affected by environmental (temperature, and water mass composition etc.) (Galand et al., 2009) and dispersal (geographic barriers) conditions (Sul et al., 2013). Metagenomic analyses of 339 environmental samples showed that environmental conditions have more influence on microbial gene pools than dispersal conditions (Fondi et al., 2016). The Arctic and Antarctic regions are geographically distant, but have similar environmental conditions (Karl, 1993; Wheeler et al., 1996). In the North and South poles, similar environments may drive similar gene pools, which could result in the observed distribution pattern of alginate utilization genes in this study.

## Conclusion

We isolated 88 strains from both polar regions, of which 60 strains could grow in the medium with alginate as the sole carbon source. These 60 strains belong to the phyla *Proteobacteria* and *Bacteroidetes*, indicating that *Proteobacteria* and *Bacteroidetes* play an important role in the degradation of alginate in polar regions. The genomic analyses of 32 alginate-utilizing strains show that 5 key enzyme genes for alginate utilization are ubiquitous in these genomes. *Proteobacteria* may have different alginate transport system from *Bacteroidetes*. Biogeographic analyses show that there is no significant difference in alginate utilization protein sequences between the Arctic and Antarctic. This implies that long-distance is not a limitation in gene transfer between Arctic and Antarctic regions. The fact that the same alginate utilization genes are widespread in both polar regions implies that the composition of alginate determines the kinds of genes required for utilization, and similar genes are required to perform the same function in similar environments. In the future, more efforts will be needed to combine metagenomics to obtain more genetic information about the marine bacteria with alginate utilization ability.

## Data Availability Statement

The datasets presented in this study can be found in online repositories. The names of the repository/repositories and accession number(s) can be found in the article/Supplementary Material.

## Author Contributions

Q-LQ designed the research. FX directed the research. Q-QC, X-JW, and X-BR performed the experiments. Q-QC, DL, PW, P-YL, H-HF, and X-YZ performed the bioinformatic analysis. Q-QC, FX, and Q-LQ wrote the manuscript. X-LC and Y-ZZ edited the manuscript. All authors contributed to the article and approved the submitted version.

## Conflict of Interest

The authors declare that the research was conducted in the absence of any commercial or financial relationships that could be construed as a potential conflict of interest.

## Footnotes

1 http://www.cazy.org/

2 http://rast.nmpdr.org/
