# Supplementary material for: Comparison of Alginate Utilization Pathways in Culturable Bacteria Isolated From Arctic and Antarctic Marine Environments
Source: Front Microbiol. 2021 Jan 27;12:609393. doi: 10.3389/fmicb.2021.609393 (PMC7874173; doi:10.3389/fmicb.2021.609393)
Supplement: Supplementary file 1 [file Data_Sheet_1.docx]

**Supplementary Table S1.** The information of sampling stations.

| Geographic location | Isolation source | Name | | Longitude and latitude |
| --- | --- | --- | --- | --- |
| Arctic | brown algae | BA1 | 78°55′15″ N, 11°57′09″ E | |
|  |  | BA2 | 78°54′57″ N, 11°59′22″ E | |
|  |  | BA3 | 78°54′58″ N, 11°59′14″ E | |
| Antarctic | penguin droppings | PD1 | 62°13′50″ S, 58°58′15″ W | |
|  |  | PD2 | 62°13′02″ S, 58°56′15″ W | |
|  | seawater | SWA1 | 62°14′05″ S, 58°54′11″ W | |
|  |  | SWA2 | 62°12′02″ S, 58°52′10″ W | |
|  | sediment | SED | 62°13′02″ S, 58°56′15″ W | |

**Supplementary Table S2.** The general characteristics for genome sequences of 26 strains sequenced in this study.

| **No** | **Strain** | **Accession number** | **Size (Mb)** | **CDSs (protein)** | **GC (%)** | **Completeness**  **(%)** | **Contamination**  **(%)** | **Geographic location** | **Isolation source** | **alginate utilization**  **genes content (%)** |
| --- | --- | --- | --- | --- | --- | --- | --- | --- | --- | --- |
| **1** | ***Polaribacter* sp. 11A2H** | **WTLD00000000** | **4** | **3294** | **30.4** | **99** | **0.5** | **Antarctic** | **penguin droppings** | **0.3** |
| **2** | ***Cellulophaga* sp. Z1A5H** | **WTLE00000000** | **5.1** | **4220** | **33.7** | **100** | **1.4** | **Antarctic** | **seawater** | **0.4** |
| **3** | ***Polaribacter* sp. 20A6** | **WTPZ00000000** | **4.6** | **3633** | **31** | **99** | **0.5** | **Antarctic** | **sediment** | **0.4** |
| **4** | ***Paraglaciecola* sp. 20A4** | **WTPY00000000** | **5.4** | **4435** | **42.6** | **100** | **0** | **Antarctic** | **sediment** | **0.3** |
| **5** | ***Pseudoalteromonas* sp. Z9A5** | **WTLC00000000** | **4.8** | **4089** | **37.9** | **100** | **0.1** | **Antarctic** | **seawater** | **0.3** |
| **6** | ***Pseudoalteromonas* sp. Z9A4** | **WTLA00000000** | **4.3** | **3721** | **39** | **100** | **0.1** | **Antarctic** | **seawater** | **0.2** |
| **7** | ***Pseudoalteromonas* sp. Z9A6** | **WTKZ00000000** | **4.5** | **3840** | **39.2** | **100** | **5** | **Antarctic** | **seawater** | **0.3** |
| **8** | ***Pseudoalteromonas* sp. Z1A8** | **WTLB00000000** | **4.4** | **3812** | **39.1** | **100** | **0** | **Antarctic** | **seawater** | **0.3** |
| **9** | ***Pseudoalteromonas* sp. 31A1** | **WTKY00000000** | **4.6** | **3900** | **39** | **100** | **0** | **Antarctic** | **penguin droppings** | **0.3** |
| **10** | ***Pseudoalteromonas* sp. Z1A2** | **WTKX00000000** | **4.5** | **3875** | **39.2** | **100** | **0.1** | **Antarctic** | **seawater** | **0.3** |
| **11** | ***Pseudoalteromonas* sp. Z1A6** | **WTKW00000000** | **4.5** | **3883** | **39.1** | **100** | **0** | **Antarctic** | **seawater** | **0.2** |
| **12** | ***Pseudoalteromonas* sp. SA25** | **WTKV00000000** | **4.8** | **4064** | **39.1** | **100** | **0** | **Antarctic** | **sediment** | **0.2** |
| **13** | ***Psychromonas* sp. SA13A** | **WTKU00000000** | **4.6** | **3780** | **37.5** | **100** | **4** | **Antarctic** | **sediment** | **0.3** |
| **14** | ***Pseudoalteromonas* sp. C8** | **WTKT00000000** | **4.5** | **3837** | **39.1** | **100** | **0.6** | **Arctic** | **brown algae** | **0.3** |
| **15** | ***Pseudoalteromonas* sp. C7** | **WTKS00000000** | **4.5** | **3867** | **39.1** | **100** | **0.8** | **Arctic** | **brown algae** | **0.3** |
| **16** | ***Pseudoalteromonas* sp. C1** | **WTKR00000000** | **4.5** | **3836** | **39.1** | **100** | **0** | **Arctic** | **brown algae** | **0.3** |
| **17** | ***Psychromonas* sp. L1A2** | **WUAG00000000** | **4.3** | **3504** | **36.3** | **100** | **1** | **Arctic** | **brown algae** | **0.4** |
| **18** | ***Colwellia* sp. 20A7** | **CP047130** | **4.5** | **3790** | **37.8** | **100** | **0.8** | **Antarctic** | **sediment** | **0.2** |
| **19** | ***Cellulophaga* sp. L1A9** | **CP047027** | **5.1** | **4334** | **34** | **100** | **1.4** | **Arctic** | **brown algae** | **0.4** |
| **20** | ***Paraglaciecola* sp. L1A13** | **CP047024** | **5** | **4075** | **42.6** | **100** | **0** | **Arctic** | **brown algae** | **0.4** |
| **21** | ***Polaribacter* sp. L3A8** | **CP047026** | **4.2** | **3410** | **30.5** | **99** | **1** | **Arctic** | **brown algae** | **0.4** |
| **22** | ***Algibacter* sp. L1A34** | **CP047029** | **4.6** | **3810** | **32.5** | **100** | **1** | **Arctic** | **brown algae** | **0.4** |
| **23** | ***Algibacter* sp. L3A6** | **CP047030** | **4.6** | **3689** | **33.3** | **99** | **0.5** | **Arctic** | **brown algae** | **0.5** |
| **24** | ***Formosa* sp. L2A11** | **CP047028** | **3.8** | **3143** | **32.2** | **99** | **0.3** | **Arctic** | **brown algae** | **0.3** |
| **25** | ***Paraglaciecola* sp. L3A3** | **CP047023** | **5.3** | **4277** | **38.6** | **100** | **2.5** | **Arctic** | **brown algae** | **0.3** |
| **26** | ***Cobetia* sp. L2A1** | **CP047025** | **4.1** | **3376** | **57.7** | **100** | **0** | **Arctic** | **brown algae** | **0.2** |

**Supplementary Table S3.** The information about six genomes downloaded from NCBI databases.

| Geographic location | Strain | Accession number | |  |
| --- | --- | --- | --- | --- |
|  |  | 16S rRNA gene | genome | |
| Arctic | *Alteromonas stellipolaris* strain LMG 21861 | NR_025433.1 | CP013926.1 | |
| Antarctic | *Arcticibacterium luteifluviistationis* strain SM1504 | NR_156148.1 | CP029480.1 | |
|  | *Polaribacter sejongensis* strain KCTC 23670 | HQ853596.1 | CP019336.1 | |
|  | *Flavobacterium faecale* strain WV33 | NR_134034.2 | CP020918.1 | |
|  | *Flavobacterium kingsejongi* strain WV39 | NR_159896.1 | CP020919.1 | |
|  | *Changchengzhania lutea* strain SM1355 | NR_159216.1 | CP039456.1 | |

**Supplementary Table S****4.** Comparison of the alginate lyase families of the different phyla.

| Phylum | Family | Count |
| --- | --- | --- |
| *Proteobacteria* | PL6 | 20 |
|  | PL7 | 38 |
|  | PL17 | 20 |
|  | PL18 | 11 |
|  | PL34 | 2 |
| *Bacteroidetes* | PL6 | 25 |
|  | PL7 | 30 |
|  | PL12 | 2 |
|  | PL17 | 11 |


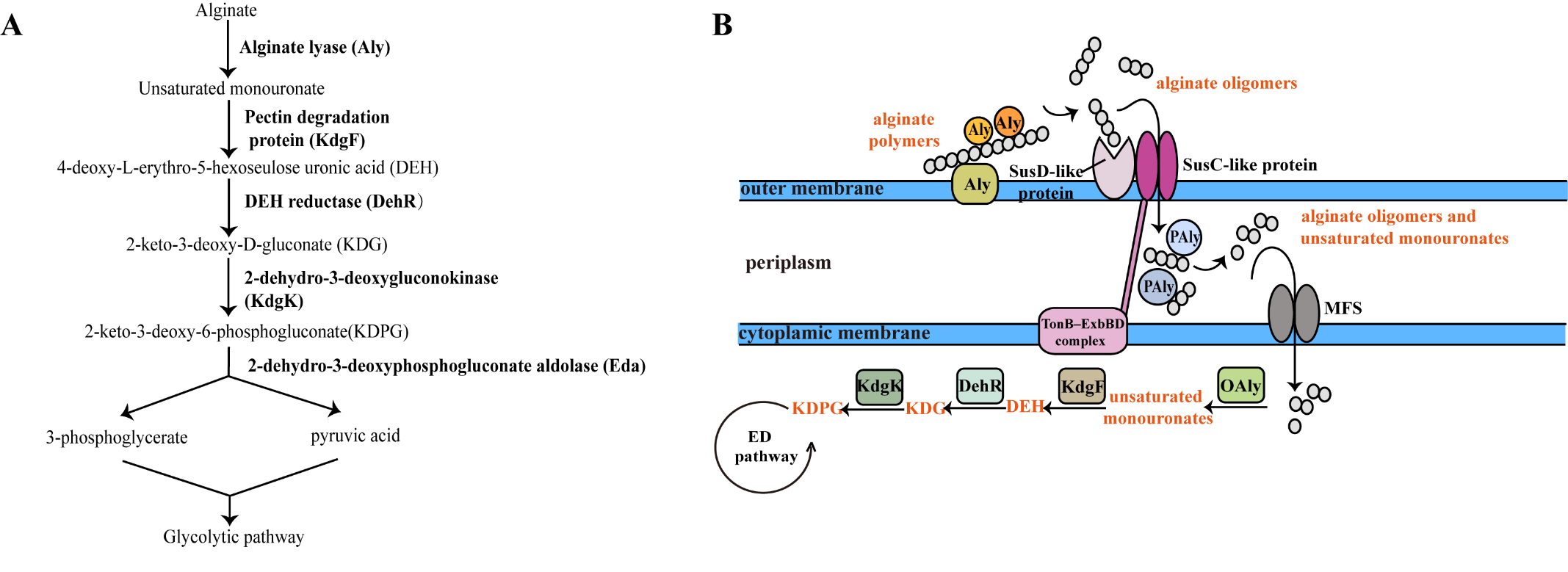


**Supplementary Figure S1.** The alginate utilization pathway (A) and the alginate transport system (B) in marine bacteria. PAly, Periplasmic Alginate lyase; OAly, Oligoalginate lyases.





**Supplementary Figure S2.** Abundances of the alginate-degrading bacteria isolated from different matrixes.


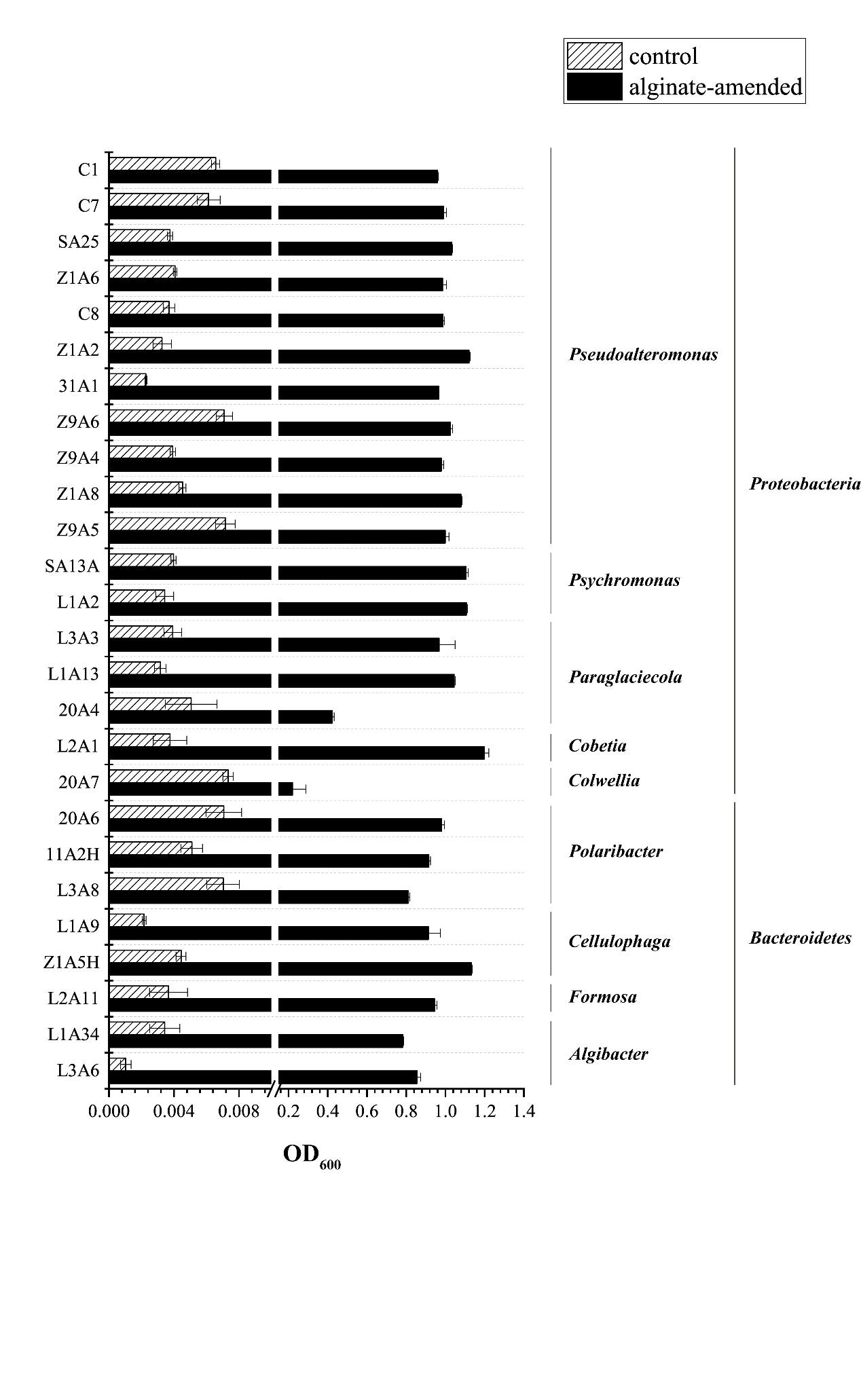


**Supplementary Figure S3.** The alginate utilization ability of 26 strains. The histograms are the growth of the 26 strains in alginate sole carbon source broth. The OD_600_ value of each strain is that at the end of the logarithmic growth period.





**Supplementary Figure S4.** The locus tags of the alginate gene cluster in Figure 3. The genes represent by “*” are annotated as hypothetical proteins in RAST, but are not annotated as any proteins in NCBI Prokaryotic Annotation Pipeline.


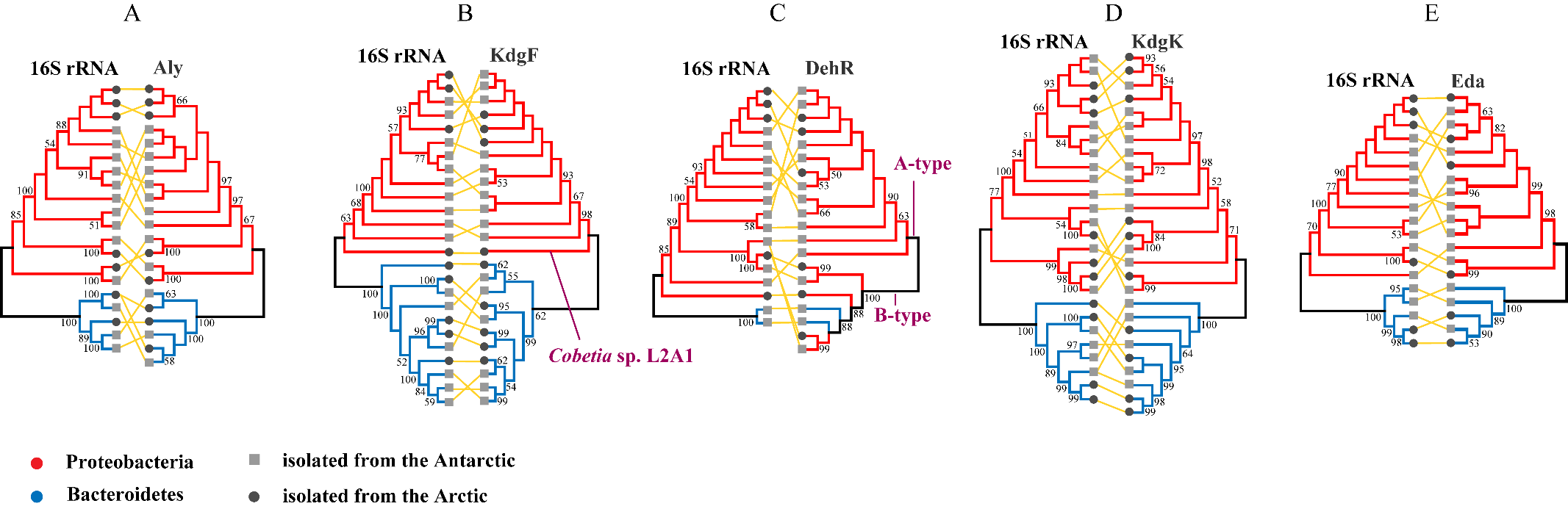


**Supplementary Figure S5.** Comparison of the maximum-likelihood phylogenetic trees constructed based on the protein sequences of 5 key enzymes of alginate utilization (the right) and the 16S rRNA gene sequences (the left). Bootstrap values (>50%) based on 1000 replicates are shown at nodes. The terminal in both trees represent the strain where the protein sequence and the 16S rRNA gene sequence are from. The same strain in both trees is linked with yellow line. The strains from *Proteobacteria* are marked in red, and the strains from *Bacteroidetes* are marked in blue. The strains isolated from Antarctic are marked in grey square, and the strains isolated from Arctic are marked in dark grey circle. The comparisons of Aly, KdgF, DehR, KdgK and Eda are marked as A, B, C, D and E, respectively.


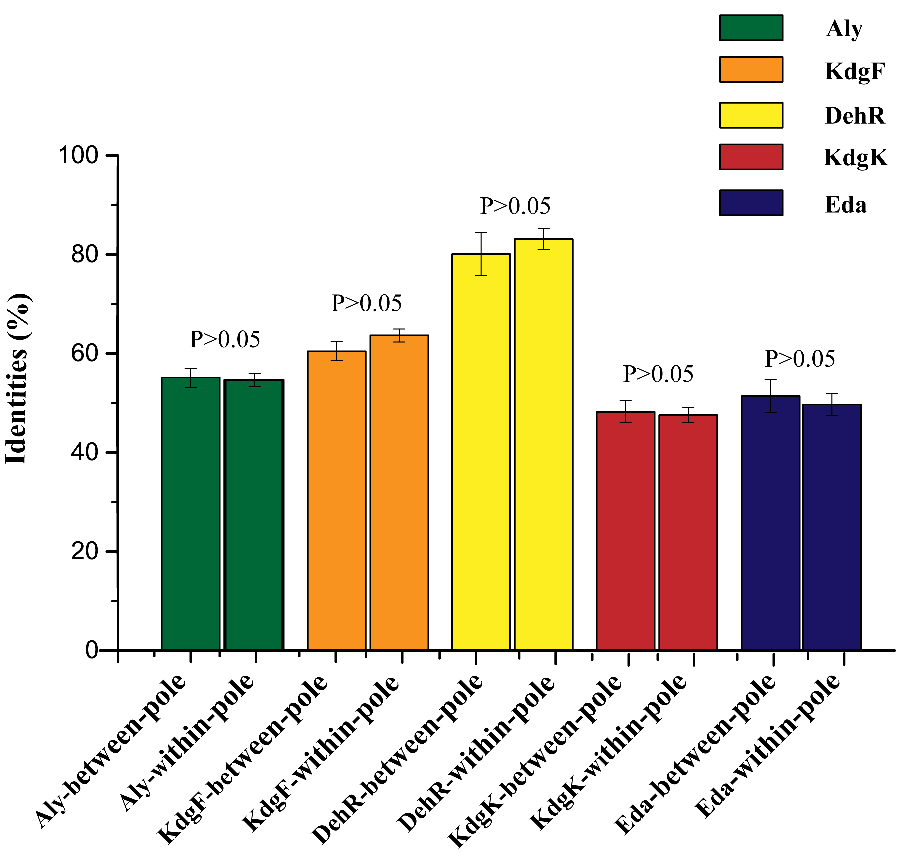


**Supplementary Figure S6.** Comparison of the between-pole and within-pole protein sequences identities of 5 key enzymes.
